# Novel therapeutic strategies targeting tumor-stromal interactions in pancreatic cancer

**DOI:** 10.3389/fphys.2013.00331

**Published:** 2013-11-11

**Authors:** Shin Hamada, Atsushi Masamune, Tooru Shimosegawa

**Affiliations:** Division of Gastroenterology, Tohoku University Graduate School of MedicineSendai, Miyagi, Japan

**Keywords:** desmoplasia, pancreatic stellate cells, cell survival, sonic hedgehog inhibitor, angiotensin receptor blocker, chemoresistance

## Abstract

Therapy-resistance and postoperative recurrence are causes of the poor prognosis in pancreatic cancer. Conventional therapies have a limited impact on the control of pancreatic cancer, resulting in the rapid re-growth of the tumor. The indispensable role of tumor-stromal interaction, which acts as a defender of cancer cells and enhances malignant potential, is being uncovered now. For example, specific signaling pathways for desmoplasia induction have been identified, such as sonic hedgehog (Shh) or connective tissue growth factor (CTGF), whose inhibition causes desmoplasia depletion and therapeutic advantages at least in *in vivo* mouse models of pancreatic cancer. Revolutions in drug delivery methods have led to the establishment of novel chemotherapeutic regimens, with better patient survival. Furthermore, mechanisms of immunosuppression in the pancreatic cancer-bearing host were clarified by the identification of myeloid-derived suppressor cells (MDSCs), which also promote disease progression. Strategies to target these components of the tumor stroma revealed certain anticancer effects *in vitro* and *in vivo*, suggesting the possibility of stroma-targeting therapy. Suppression of the stromal cell function increases the sensitivity of pancreatic cancer cells to therapeutic intervention. Further study will clarify the complex nature of the tumor microenvironment, the targeting of which has the potential to improve clinical outcome.

## Introduction

Radical surgical resection for pancreatic cancer is a curative therapy, but benefits only a small percentage (~20%) of pancreatic cancer patients. Even when such patients receive surgical resection, early recurrence and metastasis threaten their lives. Most pancreatic cancer patients show metastatic invasion to large blood vessels or distant organs, resulting in unresectable disease. The prognosis of inoperable patients is extremely poor, due to the lack of an effective therapy (Hidalgo, 2010; Michl and Gress, 2013). According to the Japan Pancreatic Cancer Registry, which summarized the clinical data of pancreatic cancer in Japan, the prognosis of pancreatic cancer overall has been improving for over 30 years, but the 5-year survival ratio is still lower than 20% (Egawa et al., 2012). The clinical problem of pancreatic cancer is its resistance to conventional therapies, such as chemotherapy or radiation. Even though these therapies reveal suppressive effects on tumor growth for a while, re-growth of the tumor frequently occurs. Furthermore, conventional chemotherapy, gemcitabine itself was shown to induce therapy-resistant populations of cancer cells in *in vivo* xenograft model in a previous study, suggesting specific mechanisms underlying the development of resistance (Jimeno et al., 2009). Therefore, an additional therapeutic strategy needs to be established to prevent the development of resistance.

Gemcitabine is a well-established therapeutic agent for unresectable pancreatic cancer, but complete remission of the disease rarely occurs (Burris et al., 1997). Gemcitabine alleviated disease-related symptoms in this study, but nearly 50% of the patients treated with gemcitabine showed only a partial response or static disease in imaging studies. Additional chemotherapeutic regimens using cytotoxic agents such as cisplatin, 5-fluorouracil (5-FU) or capecitabine in combination with gemcitabine were reported, but significant improvement in the patients' survival has not been achieved (Berlin et al., 2002; Heinemann et al., 2006; Herrmann et al., 2007). Targeted therapies were also tested alone or in combination with chemotherapy, such as vascular endothelial growth factor antibody and multikinase inhibitors. However, these agents have also failed to show improvement in patients' survival, so far (Kindler et al., 2010, 2012; O'Reilly et al., 2010). In addition, pancreatic cancer shows resistance against radiation therapy. A systematic review of the management of locally advanced pancreatic cancer demonstrated that radiation therapy alone does not have a survival benefit over that of chemoradiation therapy, suggesting the difficulties in controlling pancreatic cancer by radiation alone (Sultana et al., 2007). Recent studies indicated some beneficial effects of chemoradiation for patients with borderline resectable pancreatic cancer, but its effect on the patients with locally advanced disease remains controversial (Goodman and Hajj, 2013). Nevertheless, radiation has few benefits for metastatic pancreatic cancer.

These clinical features of pancreatic cancer have been considered to be the result of resistance in the cancer cells themselves, such as increased cell proliferation, enhanced survival signal, and blocked apoptotic pathways. Indeed, cumulative gene mutations provide these characteristics to cancer cells, which require more than 20 years for the establishment of metastatic disease (Yachida et al., 2010). However, host cells are also exposed to various signals from the pancreatic cancer cells at the same time. Recent research identified that cancer stromal cells play pivotal roles during the progression of pancreatic cancer, providing a cancer-promoting microenvironment. In pancreatic cancer tissue, cancer cells are surrounded by fibrotic stroma called desmoplasia, which sometimes occupy a larger area than cancer cells (Erkan et al., 2012). Pancreatic stellate cells (PSCs) play a central role in the formation of fibrotic tumor stroma (Apte et al., 2004; Bachem et al., 2005; Vonlaufen et al., 2008; Masamune and Shimosegawa, 2009, 2013). The interaction between cancer cells and stromal cells perpetuates inflammation within the pancreatic cancer tissue, which drives the formation and maintenance of desmoplasia. This tissue structure and extracellular matrix (ECM) proteins were reported to increase pancreatic cancer cell chemoresistance against gemcitabine and 5-FU (Erkan et al., 2007). Similarly, the ECM component hyaluronan, a megadalton glycosaminoglycan, was also reported to impair the vascular function and drug delivery in a genetically engineered mouse model of pancreatic cancer (Jacobetz et al., 2013). Another report described that the expression of Secreted protein acidic and rich in cysteine (SPARC) in the tumor stroma was inversely correlated with patients' survival. This study confirmed the invasion-promoting role of exogenous SPARC in pancreatic cancer cells, suggesting a tumor-promoting role of ECM proteins (Mantoni et al., 2008). In addition, pancreatic cancer-derived immunosuppression also contributes to the disease progression, which was confirmed by the existence of myeloid-derived suppressor cells (MDSCs) in pancreatic cancer tissue (Clark et al., 2007; Evans and Costello, 2012). These studies indicate that tumor-stromal interactions contribute to therapy-resistance in pancreatic cancer, which therefore could be an alternative therapeutic target.

Recently, attempts to treat pancreatic cancer by targeting tumor-stromal interactions have been reported. Various strategies have been examined such as targeting PSCs, inhibiting ECM deposition, suppressing angiogenesis and restoring the immune response in pancreatic cancer. Some of these strategies suggested the possibility of targeting the tumor stroma of pancreatic cancer as a novel therapeutic option. Since stromal cells maintain intact intracellular signals, these cells are assumed to show better responses to therapeutic intervention compared with cancer cells. In addition, the therapy-resistant evolution seen in cancer cells is a rare phenomenon in normal cells, based on the normal genomic regulation and lack of oncogenic mutations. The combination of novel strategies with conventional therapies should improve the clinical outcomes of pancreatic cancer. This review article summarizes the mechanisms of therapy-resistance in pancreatic cancer that are provided by tumor-stromal interactions. The current status and benefits of novel therapeutic strategies that modify drug delivery and target tumor-stromal interactions are discussed.

## Critical mediators of tumor-stromal interaction

Desmoplasia consists of the deposition of ECM proteins and consistently activated stromal cells such as PSCs and fibroblasts. Among stromal cells, PSCs play a central role in ECM production and trigger continuous inflammation through cytokine production (Erkan et al., 2012). In addition to PSCs, cancer-associated fibroblasts suppress blood vessel formation leading to the sparse vasculature, making drug delivery more difficult (Olson and Hanahan, 2009). These stromal cells contribute to the establishment of desmoplasia that involve the activation of multiple signaling pathways and cell-to-cell interactions. However, the entire picture of these processes remains ambiguous. Recent research identified some of the critical pathways that induce desmoplasia in pancreatic cancer that could be pharmaceutically targeted.

For example, sonic hedgehog (Shh) is highly expressed in pancreatic cancer tissues and their precursor lesions, which suggests some contribution to the pancreatic cancer progression (Kayed et al., 2006). The hedgehog signal plays an important role in cell-fate determination during organ development by modulating multiple cellular functions. Recently, pancreatic cancer cell-derived Shh was found to induce desmoplasia in an orthotopic implantation model of pancreatic cancer in athymic nude mice (Bailey et al., 2008). Shh affected the differentiation of human PSCs and fibroblasts, demonstrating an indispensable role as a mediator of the desmoplastic reaction. Another study identified that connective tissue growth factor (CTGF) expression was elevated in pancreatic cancer tissue compared with normal pancreatic tissue. CTGF is able to bind various growth factors or integrins modifying their activity (Abreu et al., 2002; Heng et al., 2006). CTGF was found to stimulate the proliferation of PSCs, migration, and fibrogenesis (Gao and Brigstock, 2006). CTGF expression was associated with the elevated expression of the endogenous hypoxia marker carbonic anhydrase-IX in pancreatic cancer tissue, suggesting that pancreatic cancer cell-derived factors affect the tissue structure and microenvironment (Bennewith et al., 2009).

In turn, PSCs activate multiple signaling pathways in pancreatic cancer cells. Indirect co-culture of PSCs with human pancreatic cancer cell lines activated extracellular signal-regulated kinase (ERK) and Akt pathways *in vitro*, which are cell survival-related signaling pathways (Takikawa et al., 2013). Furthermore, PSCs promote cancer metastasis in an orthotopic implantation model and increase cellular migration of cancer cells (Vonlaufen et al., 2008).

Interaction between PSCs and pancreatic cancer cells also enhanced cancer-stem cell (CSC)-related phenotypes such as increased *in vivo* tumorigenicity, the *in vitro* ability to form spheroids and epithelial-mesenchymal transition (EMT) (Kikuta et al., 2010; Hamada et al., 2012). ECM proteins produced from PSCs were also found to play cancer-promoting role by previous studies. For instance, ECM protein SPARC promoted the EMT of cancer cells (Neuzillet et al., 2013). These cell-to-cell interactions form a feed-forward loop, which perpetuates the fibrogenic process within pancreatic cancer. Therefore, the inhibition of specific pathways indispensable for desmoplasia and broad suppression of the stromal function were evaluated for therapeutic application.

## Effect of inhibiting desmoplasia-promoting pathways

Recently, the novel chemotherapeutic agent nab-paclitaxel became available for the treatment of pancreatic cancer (Von Hoff et al., 2011). This albumin-bound paclitaxel-based formula enables the hydrophobic paclitaxel to be administered at higher doses of the drug without solvent, and increases the maximum tolerated dose (Ibrahim et al., 2002). In addition, preoperative nab-paclitaxel administration decreased collagen deposition and cancer-associated fibroblasts in resected specimens, suggesting that nab-paclitaxel has an additional effect, tumor stroma disruption (Alvarez et al., 2013). Accordingly, the combination of nab-paclitaxel with gemcitabine increased the intratumoral gemcitabine concentration, leading to enhanced antitumor activity. The effect of nab-paclitaxel on the tumor stroma was also confirmed in the genetically engineered mouse model of pancreatic cancer, with impaired collagen maturation (Neesse et al., 2013b). This new regimen improved the overall survival and progression-free survival of patients with metastatic pancreatic cancer (Heinemann et al., 2013). These lines of evidence are an excellent example of the clinical benefits that can be provided by the evolution of drug delivery methods. Of note, the additional effect of nab-paclitaxel on the tumor stroma, which synergistically potentiates gemcitabine's antitumor effect, suggests the possibility of tumor-stromal interaction-targeting therapy.

Several studies reported more specific strategies directed at desmoplasia. As mentioned earlier, critical signals required for the inductions of desmoplasia have been identified. The Shh pathway plays an important role during embryonic development, and is aberrantly expressed in pancreatic cancer tissue. The Shh ligand binds to its receptor. Patched allows cell membrane-associated signal activator Smoothened to mediate the downstream signal. Small-molecule inhibitors of the Shh pathway were identified such as cyclopamine, a naturally occurring teratogenic molecule (Stanton and Peng, 2010). Additional Shh inhibitors have been identified thereafter, and their effects on desmoplasia were extensively studied. Conditional expression of mutant *K-ras* (constitutively active mutation G12D) and mutant *p53* (inactivating mutation R172H) in mice pancreas recapitulates pancreatic cancer development, which shows a progression pattern similar to that of human pancreatic cancer such as liver metastasis and desmoplasia (Hingorani et al., 2005). Pancreatic tumors in this KPC mouse were resistant to gemcitabine, as confirmed by the sustained tumor growth under gemcitabine administration (Olive et al., 2009). Isolated pancreatic cancer cells from KPC mice were sensitive to gemcitabine, and the accumulation of active metabolites of gemcitabine in isolated cancer cells was not impaired, suggesting that the tumor stroma is responsible for this resistance. Oral administration of IPI-926, a derivative of cyclopamine successfully depleted desmoplasia in the tumors of KPC mice (Olive et al., 2009). IPI-926 caused a transient increase in the vascularity of the tumors, which increased the intratumoral gemcitabine concentration. Gemcitabine or IPI-926 alone did not show survival benefits, but combination therapy with IPI-926 and gemcitabine prolonged survival. Based on these results, clinical trial using IPI-926 in combination with gemcitabine was carried out. Unfortunately, the phase II clinical trial of IPI-926 halted due to the significantly shorter survival in patients on the IPI-926 arm. This result might be due to the heterogeneity of human pancreatic cancer, which could not be recapitulated by genetically engineered mouse model. In addition, depletion of desmoplasia by a single agent might be insufficient to eliminate cancer cells completely.

An alternative pathway has also been examined as a therapeutic target. Conditional expression of mutant *K-ras* (constitutively active mutation G12D) with TGF-β receptor type II knockout in pancreas also developed pancreatic cancer in mice, accompanied by desmoplasia. Elevated expression of CTGF was detected in these pancreatic tumors, and its induction was mediated by Cxc chemokine signal. Cxc receptor inhibitor SB225002 administration alone could decrease the CTGF expression in pancreatic tumors and prolonged the survival of the cancer-bearing mice (Ijichi et al., 2011). In another study using a monoclonal antibody against CTGF (FG-3019), an enhancement of the chemotherapy response by combination therapy with gemcitabine in KPC mice was described. In this study, cytidine deaminase inhibitor administration did not show therapeutic advantages despite the elevated gemcitabine concentration within the pancreatic tumors, whereas FG-3019 monoclonal antibody could enhance the effect of gemcitabine (Neesse et al., 2013a). Since cytidine deaminase inhibitor did not alter the tumor microenvironment, the poorer response to gemcitabine and the cancer cell survival might be largely due to environmental factors derived from the desmoplasia.

These targeted therapies against tumor stroma have certain effect, but several problems need to be solved for the establishment of effective regimen. Resistance against single targeted therapy is a common phenomenon, such as the resistance against epidermal growth factor receptor tyrosine kinase inhibitor in non-small cell lung cancer (Sun et al., 2013). To overcome this resistance mechanism, downstream target of epidermal growth factor pathway, MEK inhibition was tested that induced apoptosis in epidermal growth factor receptor tyrosine kinase inhibitor-resistant lung cancer cells (Song et al., 2013). Taken together, targeted therapy against tumor stroma will also suffer from the resistance, which needs to be prevented by a combination approach.

## Targeting PSCs' function

Broad inhibition of the stromal function is an additional therapeutic strategy to attenuate tumor-stromal interactions. A wide variety of agents inhibit ECM production or the constitutive activation of PSCs. These agents include plant-derived polyphenol (ellagic acid, curcumin, and green tea polyphenol), nicotinamide adenine dinucleotide phosphate oxidase inhibitor (diphenylene iodonium and apocynin) and angiotensin II type 1 receptor blocker (ARB) (Masamune et al., 2005a,b, 2006, 2008; Sakurai et al., 2011). Treatment with these agents resulted in decreased ECM protein production such as collagen from PSCs. The attenuation of cytokine production from PSCs, inhibition of pro-inflammatory signals and reduced proliferation of PSCs were also observed by these treatments. Since these agents could be administered orally, tumor stroma-repressive effects have been well examined *in vivo* (Masamune et al., 2008; Sakurai et al., 2011).

Among these agents, ARB is an antihypertensive drug with established feasibility and safety in clinical use. An anti-inflammatory effect of ARB in the pancreas was found in one of the clinically-available ARBs, candesartan. The administration of candesartan in a rat model of chronic pancreatitis attenuated pancreatic inflammation and fibrosis (Yamada et al., 2003). Candesartan decreased the alpha-smooth muscle actin-positive cells in pancreas, suggesting suppressed activation of PSCs. A retrospective study of patients with pancreatic cancer who received angiotensin I-converting enzyme inhibitors (ACEIs) and ARBs demonstrated the contribution of both drugs to a better prognosis (Nakai et al., 2010). The overall survival was 15.1 months in the ACEI/ARB group and 8.9 months in the non-ACEI/ARB group (Nakai et al., 2010). Another report described the use of olmesartan to treat subcutaneous-tumor bearing immunodeficient mice. The growth of subcutaneous tumors derived from the co-injection of human pancreatic cancer cell line AsPC-1 with human PSC cell line was significantly suppressed by olmesartan (Masamune et al., 2013). Furthermore, olmesartan treatment attenuated the cell viability of PSCs and suppressed collagen production *in vitro*. Accordingly, decreased expression of alpha-smooth muscle actin and collagen deposition in subcutaneous tumors was confirmed, indicating an alteration of the microenvironment in the cancer tissue. In this study, delayed administration (2 weeks after the subcutaneous implantation of cancer cells with PSCs) of olmesartan also attenuated the growth of subcutaneous tumors, suggesting the contribution of PSCs to sustained tumor growth as well as successful tumor implantation. In addition to the inhibitory effect on PSCs, losartan, another ARB, reduced stromal collagen and hyaluronan that led to the reduction of solid stress and increased blood perfusion. This study suggested that ARB has a potential to remodel tumor microenvironment (Chauhan et al., 2013).

**Figure 1:**
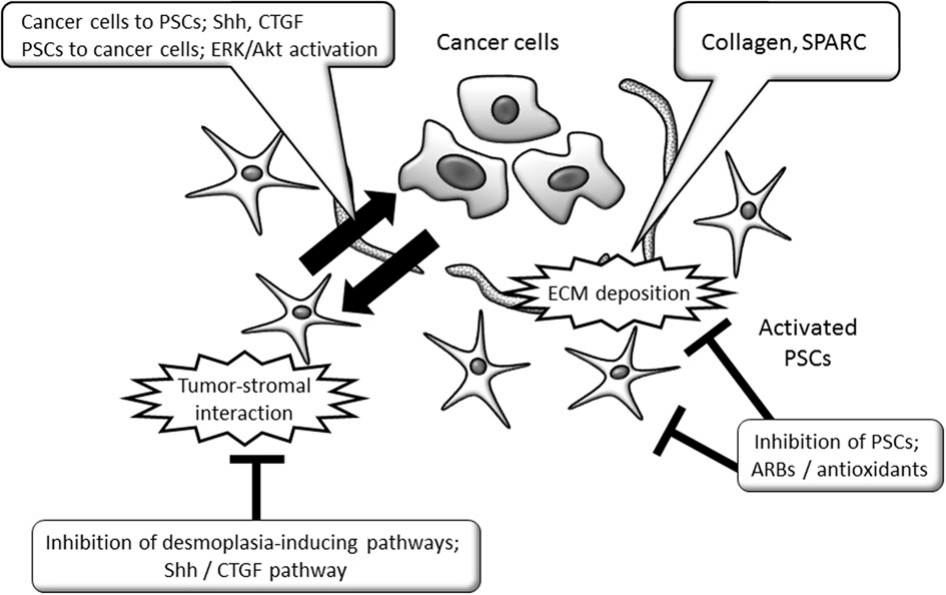
**Schematic view of tumor-promoting PSC functions and PSC-targeting strategies in pancreatic cancer.** The tumor-promoting interaction between cancer cells and PSCs could be therapeutic targets, by the inhibition of specific signaling pathways or PSC's functions. ARB, angiotensin II type 1 receptor blocker; CTGF, connective tissue growth factor; ECM, extracellular matrix; ERK, extracellular signal-regulated kinase; PSCs, Pancreatic stellate cells; Shh, sonic hedgehog; SPARC, Secreted protein acidic and rich in cysteine.

Together with the inhibition of specific signaling pathways such as Shh or CTGF, these inhibitors of PSC functions could become candidates for therapeutic applications that target tumor-stromal interaction. PSC functions that promote pancreatic cancer and possible therapeutic interventions are summarized in Figure 1.

## Modification of immune reaction against cancer cells

Interactions between pancreatic cancer cells and host immune cells play critical roles during the progression of pancreatic cancer. Recently, progress has been made in identifying the detailed mechanisms of immune suppression in pancreatic cancer. Infiltration of inflammatory cells is observed, in addition to desmoplasia, in pancreatic cancer tissue. These cells consist of immature myeloid cells that have immunosuppressive functions, known as MDSCs (Clark et al., 2007; Scarlett, 2013). These cells produce arginase, nitric oxide, and reactive oxygen species that suppress cytotoxic T-cell functions (Ostrand-Rosenberg and Sinha, 2009). Recently, several approaches have been devised to target MDSCs. The elimination of MDSCs by effector T cells targeting CD11b^+^Gr1^+^ MDSCs efficiently inhibited tumor growth (Zhang et al., 2008). Treatment with interleukin-12 was reported to attenuate the immunosuppressive effect of MDSCs by enhancing differentiation and decreasing nitric oxide synthase expression (Steding et al., 2011). Furthermore, IL-12 changed the function of MDSCs to enhance the effect of CD8^+^ T-cells, which could lead to tumor regression in the mouse model (Kerkar et al., 2011). Since MDSCs have immature nature, induction of differentiation using several reagents such as all-trans-retinoic acid or vitamin D3 was also tested (Lathers et al., 2004; Mirza et al., 2006).

Reinforcement of the host immune reaction by vaccination or dendritic cell therapy is an additional approach for modification of the immune reaction. Currently, survivin2B is targeted as a possible antigen in pancreatic cancer treatment, and immunological responses in patients have been reported (Kameshima et al., 2013). Another clinical trial targeting vascular endothelial growth factor receptor 2 by oral DNA vaccine is now in progress (Niethammer et al., 2012). As an immunotherapy, the combination of dendritic cell injection with gemcitabine administration was administered in mice that received subcutaneous implantation of pancreatic cancer cells. This treatment significantly delayed the growth of subcutaneous tumors by decreasing MDSCs within them (Ghansah et al., 2013). Previous attempts to develop cancer vaccines did not achieve favorable results, but novel targets and technical improvements might yield success. Since these therapeutic strategies targeting immune systems largely remain experimental, further examination and validation of their efficacy are required.

## Conclusion

**Figure 2:**
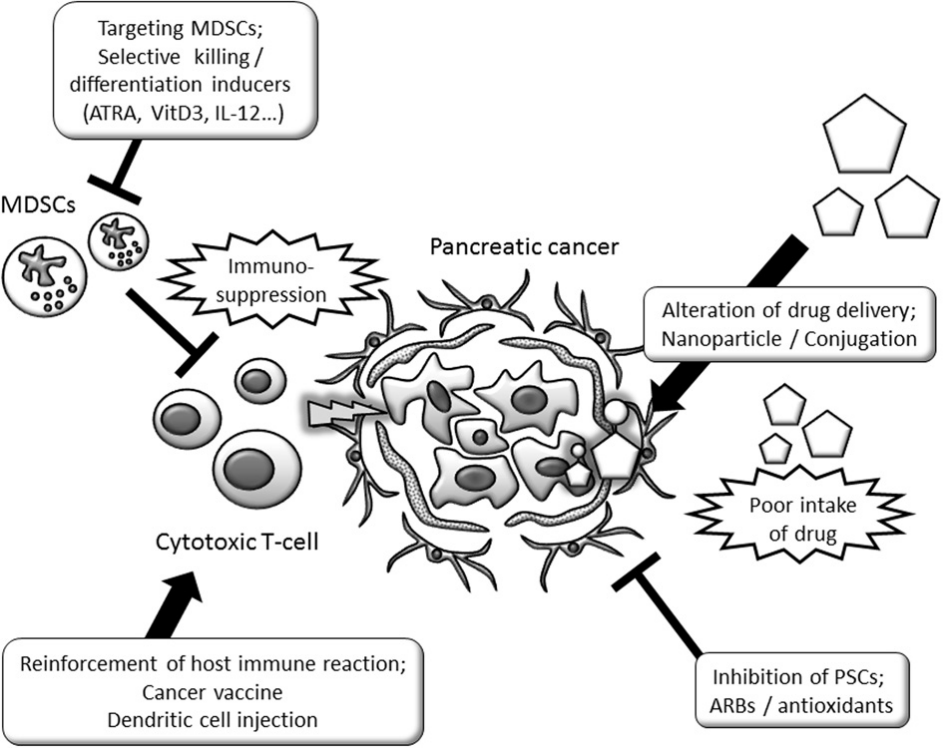
**Schematic view of therapy-resistance related tumor-stromal interaction and possibility as a tumor stroma-targeting therapy.** Stroma-targeting strategies include the modification of drug delivery, inhibition of PSC functions and restoration of immune functions. ARB, angiotensin II type 1 receptor blocker; ATRA, all-trans retinoic acid; IL, interleukin; MDSCs, myeloid-derived suppressor cells; PSCs, Pancreatic stellate cells; Shh, sonic hedgehog; SPARC, Secreted protein acidic and rich in cysteine.

Tumor-stromal interactions contribute to the specific microenvironment of pancreatic cancer and hamper effective cancer cell elimination. The relationship between tumor-stromal interaction, therapy-resistance and future perspectives for stroma-targeting therapy is summarized in Figure 2. The novel anticancer agent nab-paclitaxel provided an additional therapeutic option in pancreatic cancer treatment, with proper inhibitory effects on the tumor stroma. Present strategies for depleting the pancreatic cancer stroma itself have revealed promising effects, though their clinical application has not yet been established. Specific inducers of desmoplasia and broad inhibition of PSC functions might be combined for effective therapy. Further study will enable novel therapeutic options targeting the critical mechanisms that maintain pancreatic cancer.

### Conflict of interest statement

The authors declare that the research was conducted in the absence of any commercial or financial relationships that could be construed as a potential conflict of interest.

## Abbreviations

ACEI: angiotensin I-converting enzyme inhibitor
ARB: angiotensin II type 1 receptor blocker
CSC: cancer-stem cell
CTGF: connective tissue growth factor
ECM: extracellular matrix
ERK: extracellular signal-regulated kinase
5-FU: 5-fluorouracil
MDSCs: myeloid-derived suppressor cells
PSCs: Pancreatic stellate cells
Shh: sonic hedgehog
SPARC: Secreted protein acidic and rich in cysteine.
